# Factors Associated With Semaglutide Initiation Among Adults With Obesity

**DOI:** 10.1001/jamanetworkopen.2024.55222

**Published:** 2025-01-21

**Authors:** Meghan I. Podolsky, Rafeya Raquib, Paul R. Shafer, Katherine Hempstead, Randall P. Ellis, Andrew C. Stokes

**Affiliations:** 1 Department of Global Health, School of Public Health, Boston University, Boston, Massachusetts; 2 Now with: Bristol Meyers Squibb, Princeton, New Jersey; 3 Department of Health Law, Policy, and Management, School of Public Health, Boston University, Boston, Massachusetts; 4 The Robert Wood Johnson Foundation, Princeton, New Jersey; 5 Department of Economics, Boston University, Boston, Massachusetts

## Abstract

**Importance:**

Semaglutide, a novel glucagon-like peptide-1 (GLP-1) receptor agonist medication, was approved for weight management in individuals with obesity in June 2021. There is limited evidence on factors associated with uptake among individuals in this subgroup without diabetes.

**Objective:**

To explore factors associated with semaglutide initiation among a population of commercially insured individuals with obesity but no diagnosed diabetes.

**Design, Setting, and Participants:**

This retrospective observational cohort study used data from the Merative MarketScan Commercial Claims and Encounters Database and included adults in the US aged 18 years or older with a first diagnosis of obesity in an outpatient or inpatient setting between June 5, 2021, and July 1, 2022. Inclusion criteria were no prior antiobesity medication, GLP-1, bariatric surgery, or diabetes-related claim in the 12 months prior to obesity diagnosis, and continuous enrollment in the 12 months preceding and 6 months following obesity diagnosis. Analysis was conducted from February to November 2024.

**Exposures:**

Medication classes prescribed, clinical diagnoses, and sociodemographic factors. Exposures were identified within the 12 months prior to obesity diagnosis.

**Main Outcomes and Measures:**

Factors associated with incident semaglutide prescription within 6 months after obesity diagnoses were identified using a 10-fold cross-classified random forest model. The top 20 features of the model feature importance list were ranked in a Shapley Additive Explanations plot and used in a multivariable logistic regression model to quantify associations with semaglutide initiation.

**Results:**

In this study of 97 456 individuals, 58 124 (59.6%) were female, 26 582 (27.3%) were aged 45 to 54 years, 50 705 (52.0%) resided in the South region, and 49 390 (50.7%) were covered by preferred provider organization plans. Of all participants, 1963 (2.0%) initiated semaglutide within 6 months of their initial obesity diagnosis. The random forest model had an area under the receiver operating characteristic curve of 0.71 (95% CI, 0.69-0.74). The most important exposures identified via Shapley Additive Explanations were sex, use of antidepressants, and employer industry. The top 20 factors were used in the logistic regression model, and significant associations were found with semaglutide initiation, including being female (adjusted odds ratio [aOR], 2.30; 95% CI, 2.05-2.58), use of certain medication classes including antidepressants (aOR,1.62; 95% CI, 1.46-1.78), and being covered by a point-of-service plan (aOR, 1.78; 95% C, 1.42-2.22).

**Conclusions and Relevance:**

This cohort study found that key sociodemographic, health care, and clinical factors are associated with receipt of semaglutide in those without diabetes. These findings suggest that insurance plan type and structure may be a crucial intervention point for improving equity in obesity treatment access.

## Key Points

**Question:**

What factors are associated with semaglutide initiation in individuals with obesity who do not have diabetes?

**Findings:**

In this cohort study of 97 456 commercially insured individuals with obesity but without diabetes, 1963 (2.0%) initiated semaglutide within 6 months after documentation of an obesity diagnosis. Sex, insurance plan type, and use of common medications were associated with semaglutide initiation.

**Meaning:**

This cohort study found that sociodemographic, health care, and clinical factors were associated with receipt of effective obesity treatment, indicating potential targets for improving equity in treatment access.

## Teaser

This cohort study analyzes associations of sociodemographic, clinical, and health care factors with initiation of semaglutide among individuals with obesity without diabetes.

## Introduction

Demand for novel glucagon-like peptide-1 (GLP-1) receptor agonist medications like semaglutide (eg, Wegovy or Ozempic) and tirzepatide (eg, Zepbound or Mounjaro) has quickly escalated. Approximately 6% of the US population reports currently taking a GLP-1 receptor agonist, but the proportion among individuals who report being told by a physician that they have overweight or obesity is closer to 1 in 5.^1^ In the US, 73.6% of the population has overweight or obesity.^2^ Historically, GLP-1 receptor agonists were exclusively approved to treat type 2 diabetes, but recent clinical trials concluded that they are highly effective at weight management and cardiovascular disease prevention, leading to US Food and Drug Administration (FDA) approvals for these indications.^3^

Individuals with obesity but without diabetes represent a unique population in the context of metabolic health. This subgroup, while sometimes characterized as metabolically healthy due to the lack of prevalent disease, is at elevated risk of developing diabetes and other cardiometabolic conditions compared with individuals without obesity.^4,5,6,7^ They may be undertreated due to clinician and patient perceptions of risk and are often left behind in the metabolic care paradigm that focuses on obesity as a risk factor, not a disease.^8,9^ Individuals with concurrent dyslipidemia and hyperglycemia are more likely to receive lifestyle counseling compared with those with obesity alone.^10^ This treatment paradigm appears to persist despite the approval of the novel GLP-1 receptor agonists for obesity, with only 25% of patients obtaining payer coverage for new Wegovy prescriptions, which is indicated for weight management, compared with nearly triple the success rate for Ozempic, which is indicated for diabetes.^11^

While antiobesity medications have the potential to reduce inequities in obesity burden and treatment, they may not be reaching subpopulations disproportionately impacted by the disease, such as those with low socioeconomic status and Black and Hispanic individuals.^12,13,14^ Patient access to GLP-1 receptor agonists may vary by individual factors; one key determinant is insurance coverage, and there is scant data on variations in access by insurance source and structure.^15^ One study^16^ found that the majority of first-time users of semaglutide and tirzepatide in a collective of health care systems that provide 18% of daily clinical care in the US were White, female, and high-income, which aligns with pre–novel GLP-1 receptor agonist research in 3 integrated delivery systems (Kaiser Permanente, HealthPartners, and Denver Health) and the Veterans Health Administration, which found disparities in access to older antiobesity medications by race, gender, and presence of metabolic comorbidities and the high cost of the medications.^17,18^ However, there is limited evidence on variations in access to treatment by diabetes diagnosis. Characterizing current users of these medications without diabetes will aid in understanding how to provide effective obesity care to those in need.

In the present study, we examined factors associated with semaglutide initiation among a population of commercially insured individuals with obesity but no diagnosed diabetes. We implemented a machine learning approach to factor identification, followed by multivariable regression modeling to quantify the association of the top factors with semaglutide initiation.

## Methods

Data used in this cohort study were part of the Merative MarketScan Commercial Claims and Encounters database (formerly IBM Marketscan) from June 5, 2020, through December 31, 2022. MarketScan is a commercial health insurance claims database containing information on enrollment, demographic characteristics, inpatient and outpatient claims, and fills for prescribed medicines for insurees aged 18 to 64 years. This study was deemed exempt from review and the requirement of informed consent by the institutional review board at Boston University. This study followed the Strengthening the Reporting of Observational Studies in Epidemiology (STROBE).^19^

### Study Criteria

We sought to examine factors associated with incident semaglutide usage among individuals with obesity without diabetes, as summarized in eFigure 1 in Supplement 1. We created a cohort of individuals who had at least 1 encounter with a health care professional for obesity in an inpatient or outpatient setting, denoted as a claim with an associated *International Statistical Classification of Diseases and Related Health Problems, Tenth Revision (ICD-10)* code of E66.x. The first claim in the database was used as the baseline date of obesity diagnosis on or after June 5, 2021. Given that billing guidelines mandate that an accompanying Z68.x *ICD-10* code be used with E66.x (overweight and obesity) for incident diagnoses of obesity, we excluded individuals who did not have a Z68.x code indicating a body mass index (BMI; calculated as weight in kilograms divided by height in meters squared) of 30 or greater at their date of obesity diagnosis. If an individual had claims in both inpatient and outpatient settings for obesity, the earliest was taken as the baseline date. We then excluded individuals without 12 months of continuous enrollment prior to the baseline date and 6 months of continuous enrollment following the baseline date. Individuals with bariatric surgery, a diabetes-related visit (Clinical Classifications Software Refined [CCSR] version 2024.1, categories END002-END006), or a prescription for an antihyperglycemic agent (Red Book 2022 release [Truven Health Analytics Inc] therapeutic class 085, 172, 173, 174, 266, 267, or 268) during the 12 months prior to obesity diagnosis date were excluded from the sample. Individuals with a pregnancy-related claim during the 12 months prior to obesity diagnosis or 6 months following obesity diagnosis were excluded, as well as those with a point-of-service with capitation plan or aged 65 years at diagnosis date. Because the primary outcome was prescription of semaglutide within 6 months after the first obesity-related encounter, we only included patients whose obesity diagnosis date was between June 5, 2021, and June 30, 2022, because semaglutide was first approved for weight management on June 4, 2021, and excluded those with an obesity-related claim in the 12 months prior to their obesity diagnosis date in the identification period.^20^ While tirzepatide was approved by the FDA for the treatment of diabetes in May 2022, we did not observe any prescriptions in the database. Six months was selected as the follow-up duration due to the decline in prescribing observed after the initial postdiagnosis period (eFigure 2 in Supplement 1).

### Exposure Identification

Exposures were established during the 12-month period prior to the first obesity-related encounter. Clinical risk factors were identified by inpatient or outpatient visits with an associated *ICD-10* code and classified using CCSR categories.^21^ Pharmaceutical exposures were identified as prescribed using their national drug code number as classified into therapeutic class by Red Book. Sociodemographic factors were extracted at baseline date.

### Statistical Analyses

To identify factors associated with semaglutide use, we used 10-fold cross-validated random forest models from the Python library RandomForestClassifier, examining 698 potential variables. We used a machine learning approach to covariate selection for the logistic regression model to ensure that potentially impactful factors were not excluded. A total of 480 CCSR clinical categories, 190 therapeutic classes, and 28 demographic exposures were included (eTable 1 in Supplement 1). Ten observations was the minimum requirement for terminal nodes and splitting. The dataset was separated into 10 independent partitions, and a random forest of 1000 trees was implemented in each of the folds. Model performance was evaluated using the average area under the receiver operating characteristic curve (AUROC; a measure of how well the model ascertains whether different characteristics affect an individual’s propensity to be prescribed semaglutide). We used the model’s feature importance list to identify the top 20 features and then ranked their Shapley Additive Explanation (SHAP) values.^22^ Robustness checks were performed to evaluate the effect of outcome imbalance on the results.^23^

Once the top features were identified, a multivariable logistic regression model was implemented with the top 20 most impactful factors^24^ to ascertain the magnitude of the association between each exposure and semaglutide initiation. We retained the top 20 most impactful factors in the regression model to limit the risk of overadjustment. The threshold for statistical significance was a 2-sided *P* < .05. Statistical analysis was conducted from February to November 2024 using Stata 17 MP (StataCorp), SAS version 9.4 (SAS Institue) and Python versopm 3.10.12 (Python Software Foundation).

## Results

Of the 97 456 individuals that met inclusion criteria (eFigure 3 in Supplement 1), 58 124 (59.6%) were female, 26 582 (27.3%) were aged 45 to 54 years, 50 705 (52.0%) resided in the South region, and 49 390 (50.7%) were covered by preferred provider organization (PPO) plans (Table). Of all participants, 1963 (2.0%) initiated semaglutide within 6 months of their initial obesity diagnosis. Proportionally fewer individuals aged 18 to 24 and 55 to 64 years initiated semaglutide, comprising only 3.4% (66 individuals) and 15.7% (309 individuals) of those that initiated, respectively. The highest BMI category (≥40) comprised the largest portion of those that initiated semaglutide (796 individuals [40.6%]).

**Table.** Descriptive Characteristics of the Sample

Characteristic	Participants, No. (%)		
	Did not initiate semaglutide (n = 95 493)	Initiated semaglutide (n = 1963)	Full sample (N = 97 456)
Age group, y			
18-24	7509 (7.9)	66 (3.4)	7575 (7.8)
25-34	17 529 (18.4)	313 (15.9)	17 842 (18.3)
35-44	24 889 (26.1)	603 (30.7)	25 492 (26.2)
45-54	25 910 (27.1)	672 (34.2)	26 582 (27.3)
55-64	19 656 (20.6)	309 (15.7)	19 965 (20.5)
Sex			
Male	38 935 (40.8)	397 (20.2)	39 332 (40.4)
Female	56 558 (59.2)	1566 (79.8)	58 124 (59.6)
Region			
Northeast	11 466 (12.0)	362 (18.4)	11 828 (12.1)
North Central	22 488 (23.5)	395 (20.1)	22 883 (23.5)
South	49 610 (52.0)	1095 (55.8)	50 705 (52.0)
West	11 929 (12.5)	111 (5.7)	12 040 (12.4)
Plan type			
Basic or major medical or comprehensive	2781 (2.9)	74 (3.8)	2855 (2.9)
Exclusive provider organization or health maintenance organization	7216 (7.6)	174 (8.9)	7390 (7.6)
Point of service	12 788 (13.4)	182 (9.3)	12 970 (13.3)
Preferred provider organization	48 383 (50.7)	1007 (51.3)	49 390 (50.7)
Consumer-directed health plan	11 966 (12.5)	282 (14.4)	12 248 (12.6)
High deductible health plan	10 975 (11.5)	223 (11.4)	11 198 (11.5)
Missing	1384 (1.4)	21 (1.1)	1405 (1.4)
Industry			
Manufacturing, durable goods	15 541 (16.3)	317 (16.1)	15 858 (16.3)
Manufacturing, nondurable goods	3532 (3.7)	126 (6.4)	3658 (3.8)
Transportation, communications, or utilities	8708 (9.1)	182 (9.3)	8890 (9.1)
Retail trade	2999 (3.1)	57 (2.9)	3056 (3.1)
Finance, insurance, or real estate	6705 (7.0)	196 (10.0)	6901 (7.1)
Services	14 868 (15.6)	468 (23.8)	15 336 (15.7)
Other	1414 (1.5)	30 (1.5)	1444 (1.5)
Unknown	41 726 (43.7)	587 (29.9)	42 313 (43.4)
Body mass index category^a^			
30.0-34.9	38 349 (40.2)	593 (30.2)	38 942 (40.0)
35.0-39.9	25 812 (27.0)	574 (29.2)	26 386 (27.1)
≥40.0	31 332 (32.8)	796 (40.6)	32 128 (33.0)

^a^ As indicated by a Z68.x code from the *International Statistical Classification of Diseases and Related Health Problems, Tenth Revision (ICD-10)*; body mass index is calculated as weight in kilograms divided by height in meters squared.

**Figure 1.**
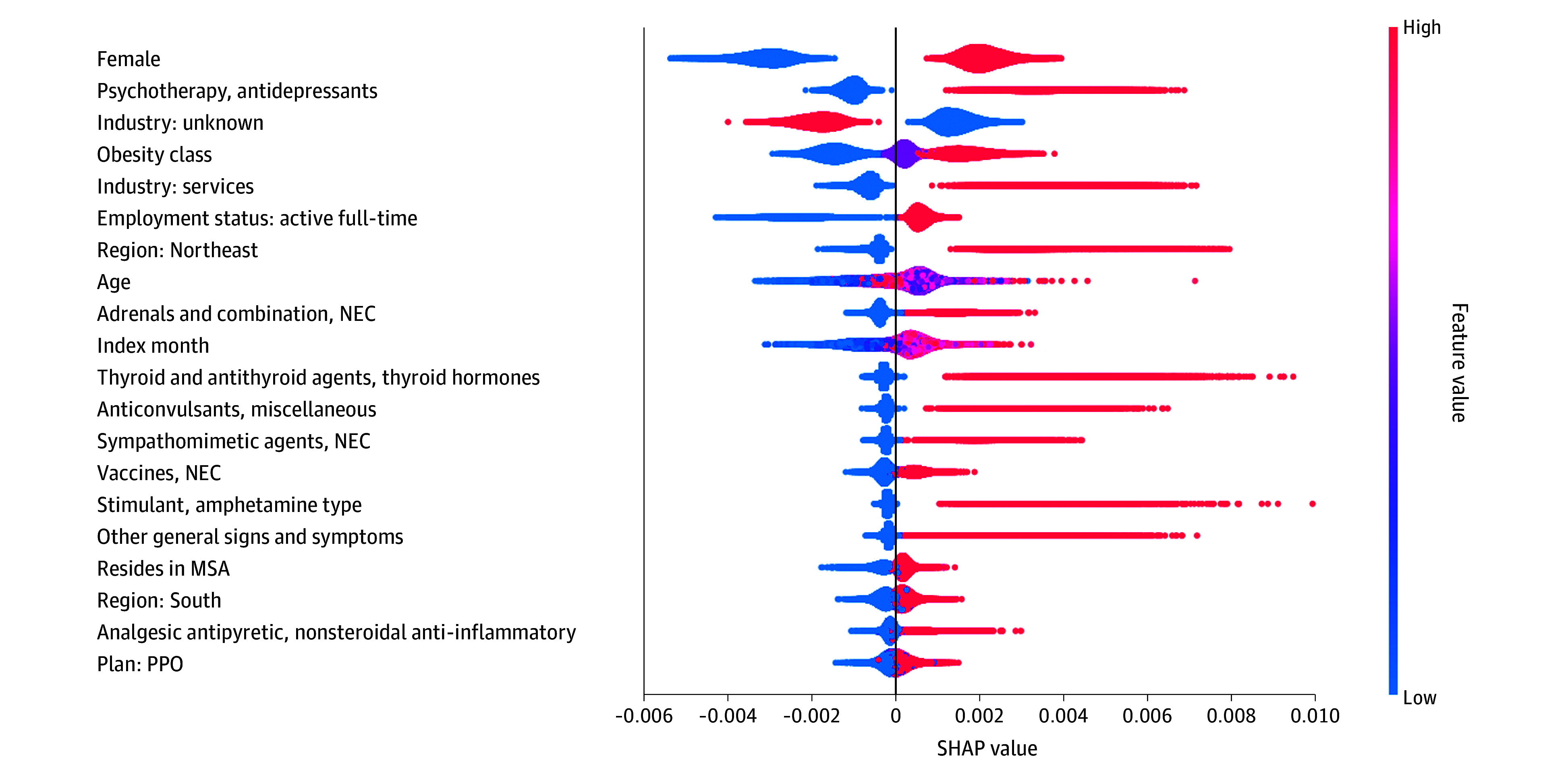
Mean Shapley Additive Explanation (SHAP) Value Summary From Cross-Classified Random Forest Model Estimating Semaglutide Initiation Among Individuals With Obesity Without Diabetes SHAP value plots for each feature are shown, ranked by importance (mean absolute SHAP value). Each dot represents 1 individual, with their position representing their SHAP value. Color is used to indicate the specified feature characteristic. Red dots indicate those who have the listed characteristic, while blue dots indicate those who do not. For example, for the characteristic female, individuals with a red dot are female and appear predominantly to the right, indicating that they are more likely to initiate semaglutide, while those with a blue dot are male. Age is a continuous variable representing age at diagnosis (18 to 64 years) and index month ranged from June 2021 to June 2022. MSA indicates metropolitan statistical area; NEC, not elsewhere classified; PPO, preferred provider organization.

eFigure 4 in Supplement 1 presents the random forest classifier model performance statistics. The AUROC was 0.71 (95% CI, 0.69-0.74), indicating that the model had reasonable discriminative performance. The top 20 features identified by the model are ranked by mean SHAP values in Figure 1. The identified features were similar in the sensitivity analysis evaluating the robustness of the results-to-outcome imbalance (eFigure 5 and eFigure 6 in Supplement 1). The factor with the highest SHAP value for semaglutide initiation was sex (mean SHAP, 0.00249), with female individuals being more likely to be classified as initiating semaglutide (eTable 2 in Supplement 1). Use of common medication classes such as antidepressants (mean SHAP, 0.00162), thyroid or hormone medications (mean SHAP, 0.00055), amphetamines (mean SHAP, 0.00033), anticonvulsants (mean SHAP, 0.00044), and adrenal medications (mean SHAP, 0.00061) were identified as important in the model. Demographic and economic factors, including age, region, and insurance plan type, as well as employee status (mean SHAP, 0.00095) and index month (mean SHAP, 0.00057) were also identified as important. Specifically, older individuals (mean SHAP, 0.00067), those in the Northeast (mean SHAP, 0.00078) and South (mean SHAP, 0.00026), those covered under a PPO plan (mean SHAP, 0.00022), and those employed in the service industry (mean SHAP, 0.00112) were more likely to be classified as initiating semaglutide, while those with unknown employer industry were less likely to be classified as initiating semaglutide.

**Figure 2.**
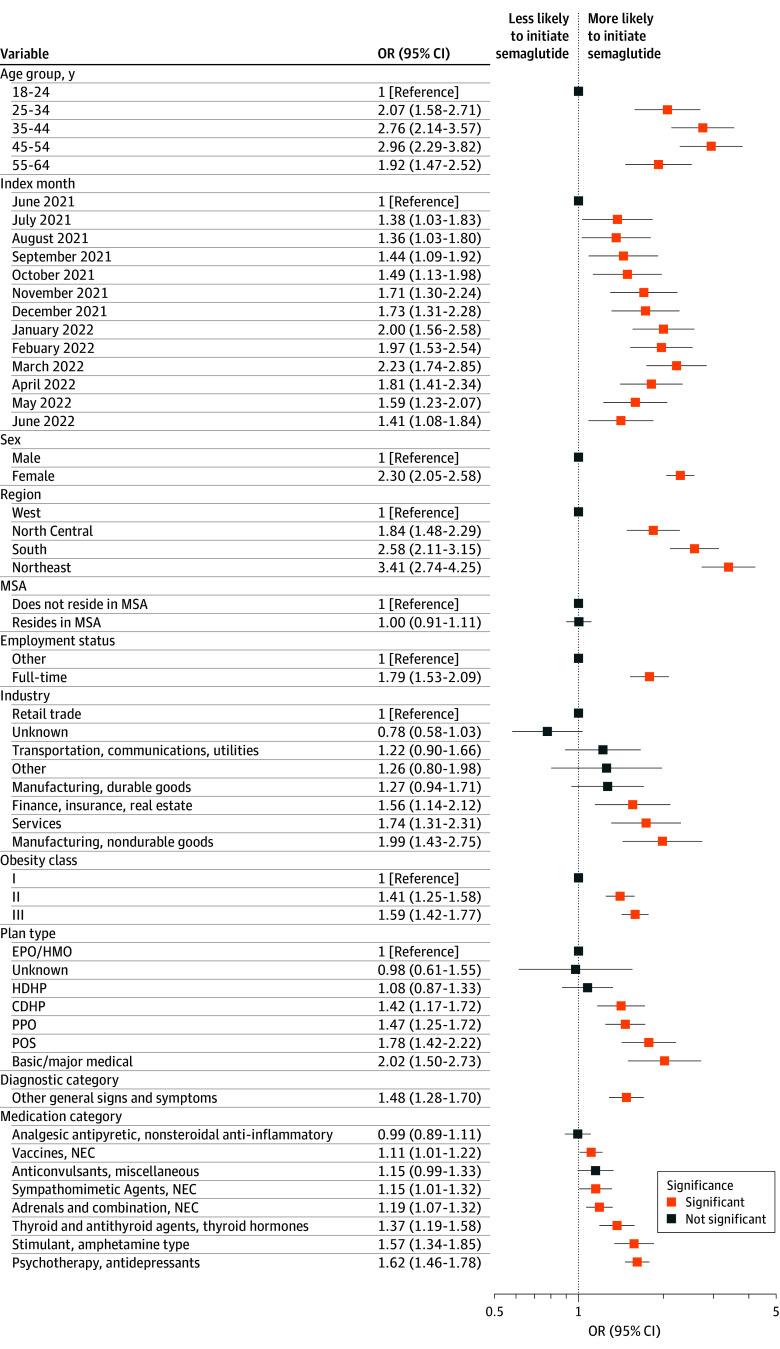
Adjusted Odds Ratios (ORs) of Top Features for Semaglutide Initiation CDHP indicates consumer-directed health plan; EPO, exclusive provider organization; HDHP, high deductible health plan; HMO, health maintenance organization; MSA, metropolitan statistical area; NEC, not elsewhere classified; POS, point of service; PPO, preferred provider organization.

Results from the multivariable logistic regression quantifying the association of the top 20 features with semaglutide initiation are shown in Figure 2. Those with more recent index dates compared with June 2021 had higher odds of initiating semaglutide within 6 months, peaking in March 2022 (adjusted odds ratio [aOR], 2.23; 95% CI, 1.74-2.85), then declining through June 2022 (aOR, 1.41; 95% CI, 1.08-1.84). Individuals in the North Central (aOR, 1.84; 95% CI, 1.48-2.29), South (aOR, 2.58; 95% CI, 2.11-3.15), and Northeast (aOR, 3.41; 95% CI, 2.74-4.25) regions were significantly more likely to initiate semaglutide compared with those residing in the West. Females had more than twice the odds of initiating semaglutide compared with males (aOR, 2.30; 95% CI, 2.05-2.58). Those whose employer was in the nondurable goods manufacturing (aOR, 1.99; 95% CI, 1.43-2.75); finance, insurance, and real estate (aOR, 1.56; 95% CI, 1.14-2.12); or services industry (aOR, 1.74; 95% CI, 1.31-2.31) were more likely to start semaglutide. Initiation varied by plan type, with individuals covered by point-of-service (aOR, 1.78; 95% CI, 1.42-2.22) and PPO plans (aOR, 1.47; 95% CI, 1.25-1.72) having higher odds of initiating compared with those covered by exclusive provider organization or health maintenance organization plans. Finally, individuals prescribed amphetamine stimulant (aOR, 1.57; 95% CI, 1.34-1.85), thyroid (aOR, 1.37; 95% CI, 1.19-1.58), and antidepressant (aOR, 1.62; 95% CI, 1.46-1.78) medications were more likely to initiate semaglutide.

## Discussion

In this cohort study, our analysis of a large commercial health insurance claims database identified several key sociodemographic, health care, and clinical factors that were associated with semaglutide initiation in individuals with obesity and without diabetes. Certain medication classes, age, sex, industry types, and plan structures were associated with semaglutide uptake.

The subgroup of individuals with obesity but without diabetes has been often omitted from discussions around equity in obesity treatment. Individuals that have metabolically healthy obesity still exhibit an elevated risk of developing cardiometabolic diseases.^25^ Older paradigms of obesity treatment conceptualized obesity as a risk factor for further metabolic disease, and while obesity does increase risk, it is now considered to be an independent disease. However, clinicians greatly underprescribe antiobesity medications compared with antidiabetic medications.^26^ Additionally, individuals with higher reported BMI at baseline were more likely to initiate semaglutide, a finding that reflects the disconnect between clinician behavior and treatment guidelines. While antiobesity medications are recommended for individuals with a BMI of 30 or greater or a BMI of 27 with a weight-related comorbidity,^27^ many clinicians opt to restrict treatment to lifestyle and behavioral therapy if an individual has a BMI less than 35. This trend persists, despite the growing body of evidence concluding that individuals with class I obesity often develop class II and III obesity over the life course.^28^

Use of other medications was found to be a significant factor associated with semaglutide uptake. Antidepressant use is common in individuals with obesity because there is a bidirectional biological association of depression with obesity.^29^ Many medications in this class have a weight-inducing effect, and one study^30^ found that 10% to 20% of individuals with obesity and diabetes were using at least 1 nondiabetes weight-inducing medication. Treatment guidelines recommend optimizing care to support weight loss among individuals with obesity, and medication selection can play a key role in facilitating or hindering this outcome. A post hoc subgroup analysis from the Semaglutide Treatment Effect for People With Obesity trial^31^ found that antidepressant use at baseline did not affect the efficacy or adverse effect profile of the medication, but further analyses are needed to determine whether specific medications may impede weight loss.

Supply shortages have also presented a major barrier to access for individuals prescribed a GLP-1 receptor agonist.^32^ In our model, those with more recent index dates were increasingly likely to receive the medication, but the trend appeared to start tapering off after March 2022, approximately when the shortages were first documented.^33^ While Novo Nordisk has pledged to improve manufacturing capacity to meet the increasing demand, as of June 2024 the lower starting doses of semaglutide (0.25, 0.5, and 1.0 mg) were still being limited to preserve continuity of care for those already taking the medication.^34^ This has led to individuals seeking out compounded versions of the drug or pursuing tirzepatide, although there have been reported shortages of this medication as well.^35,36^

We found that individuals covered by exclusive provider organization and HMO plan types were less likely to initiate semaglutide therapy. HMO plans require patients to select a primary care physician, which may result in increased utilization of care. Patient financial responsibility depends on the actuarial value and benefit design of the insurance plan.^37^ Some patients may face relatively high copay or coinsurance payments until their deductible or out-of-pocket maximum is met. Some patients may seek prescriptions even though GLP-1 receptor agonists are not covered by their insurance, in which case they would be responsible for the entire payment. These characteristics may be underlying the observed trends in semaglutide uptake. Coverage for antiobesity medications varies greatly by source of insurance and plan type, and plans may impose step therapy or behavioral modification program requirements.^38,39^ Approximately 1 in 4 employers report offering coverage for GLP-1 receptor agonists for weight loss indications, while more than three-fourths cover them for diabetes.^40,41,42^ Future analyses should investigate how plan structures can affect access to antiobesity medications and improve employee awareness of these plan dynamics prior to open enrollment periods.

Employer industry type was associated with access to semaglutide as well, which may be due to the relative earnings in different lines of work. According to the Bureau of Labor Statistics, as of April 2024 individuals employed in the financial and professional services industries earned $45.30 and $41.82 per hour, while those in the retail industry earned $24.25 per hour.^43^ Both semaglutide and tirzepatide have high list prices, hovering around $1000 per month.^44^ Manufacturer coupons can reduce the cost burden by $225 to $500 per month for patients paying out of pocket.^45^ Prices appear to be elevated for those accessing the drugs for nondiabetes indications, with the estimated monthly price net of discounts for semaglutide for diabetes being $344 to $411 lower than that of semaglutide for weight management.^46,47^ For those on public payer plans, which represents those particularly at-risk of mortality and morbidity from obesity,^48^ coverage is highly variable. Historically, Medicare Part D plans were forbidden from covering pharmacotherapies for weight management indications, while being permitted to cover those same active ingredients to treat diabetes. While policy changes have been proposed to expand access,^49^ there is still pressure to exclude these medications for budgetary concerns. However, these estimates have ignored potential long-term morbidity, mortality, and health care spending benefits,^50,51,52^ as well as the price drops that accompany new therapeutic entrants, patent expiry, and the planned Medicare drug price negotiations.^53,54^

Our findings may help clinicians better understand which patients are less likely to access obesity treatment, enabling more equitable care. For policymakers, the results underscore the need to address structural barriers, such as expanding insurance coverage of weight loss medications to promote access to effective obesity treatments. Overall, this study highlights the need to investigate antiobesity medication use in varied populations as the obesity treatment landscape changes with the introduction of novel treatments.

### Limitations

One limitation of this study is its reliance on claims data, which lags behind prescribing and health care provision by 3 to 6 months while claims are adjudicated. Therefore, the most recent data may have been missing prescriptions. Claims data do not contain the examination and laboratory data present in electronic health record data, factors which may impact obesity treatment access. Future research should leverage data sources containing these additional elements, as well as those reflecting social determinants of health and physician characteristics to further illuminate potential inequities in treatment access. Additionally, while the majority of GLP-1 receptor agonist prescribing occurs within the first 6 months after an individual’s first obesity-related visit, medication shortages, step therapy requirements, and prior authorizations may delay prescription date. We were unable to discern whether medications were prescribed but not filled. While clinical care guidelines recommend that individuals with diabetes take antihyperglycemic agents and see their clinicians regularly, those with lower adherence to recommendations may not have seen their clinician or have been prescribed an antihyperglycemic agent within the 12 months prior to their first obesity-related visit. Claims may underreport true obesity prevalence, and our analysis is limited to those whose physicians decided to submit a code during the claim process to signify treatment, which may limit generalizability and impact the factors associated with semaglutide use.

## Conclusions

In this retrospective observational cohort study of commercially insured individuals with obesity and without diabetes, we identified sociodemographic, health care, and clinical factors associated with semaglutide initiation within 6 months after obesity diagnosis using a novel machine learning approach. The associations of these factors with semaglutide initiation were quantified using multivariable logistic regression, and use of common medications, insurance plan structure, employer industry type, and sex were all significantly associated with semaglutide initiation. These findings suggest that inequities persist in medication access in this understudied subgroup, and further research should investigate factors associated with GLP-1 receptor agonist use in those with public payer plans, as well as whether concurrent use of common medications impacts effectiveness.

## Notes

Supplement 1. **eFigure 1.** Study Diagram **eFigure 2.** Trends in Semaglutide Initiation After Initial Obesity Diagnosis **eTable 1.** List of Factors Included in Model **eFigure 3.** CONSORT Diagram **eFigure 4.** Performance of 10-Fold Cross-Classified Random Forest Models for Estimating Semaglutide Initiation Among Individuals With Obesity Without Diabetes **eFigure 5.** SHAP Plot for Dataset With Oversample of Positive Class **eFigure 6.** SHAP Plot for Dataset With Oversample of Negative Class **eTable 2.** Mean and Scaled SHAP Values

Supplement 2.
Data Sharing Statement
