# Supplementary material for: Factors Associated With Semaglutide Initiation Among Adults With Obesity
Source: JAMA Netw Open. 2025 Jan 21;8(1):e2455222. doi: 10.1001/jamanetworkopen.2024.55222 (PMC11751746; doi:10.1001/jamanetworkopen.2024.55222)
Supplement: Supplement 1. — eFigure 1. Study Diagram eFigure 2. Trends in Semaglutide Initiation After Initial Obesity Diagnosis eTable 1. List of Factors Included in Model eFigure 3. CONSORT Diagram eFigure 4. Performance of 10-Fold Cross-Classified Random Forest Models for Estimating Semaglutide Initiation Among Individuals With Obesity Without Diabetes eFigure 5. SHAP Plot for Dataset With Oversample of Positive Class eFigure 6. SHAP Plot for Dataset With Oversample of Negative Class eTable 2. Mean and Scaled SHAP Values [file jamanetwopen-e2455222-s001.pdf]

Supplemental Online Content

Podolsky MI, Raquib R, Shafter PR, Hempstead K, Ellis RP, Stokes AC. Factors associated with semaglutide initiation among adults with obesity. *JAMA Netw. Open*. 2025;8(1):e2455222. doi:10.1001/jamanetworkopen.2024.55222

eFigure 1. Study Diagram

eFigure 2. Trends in Semaglutide Initiation After Initial Obesity Diagnosis

eTable 1. List of Factors Included in Model

eFigure 3. CONSORT Diagram

eFigure 4. Performance of 10-Fold Cross-Classified Random Forest Models for Estimating Semaglutide Initiation Among Individuals With Obesity Without Diabetes

eFigure 5. SHAP Plot for Dataset With Oversample of Positive Class

eFigure 6. SHAP Plot for Dataset With Oversample of Negative Class

eTable 2. Mean and Scaled SHAP Values

This supplemental material has been provided by the authors to give readers additional information about their work.

© 2025 Podolsky MI et al. JAMA Network Open.

eFigure 1. Study Diagram

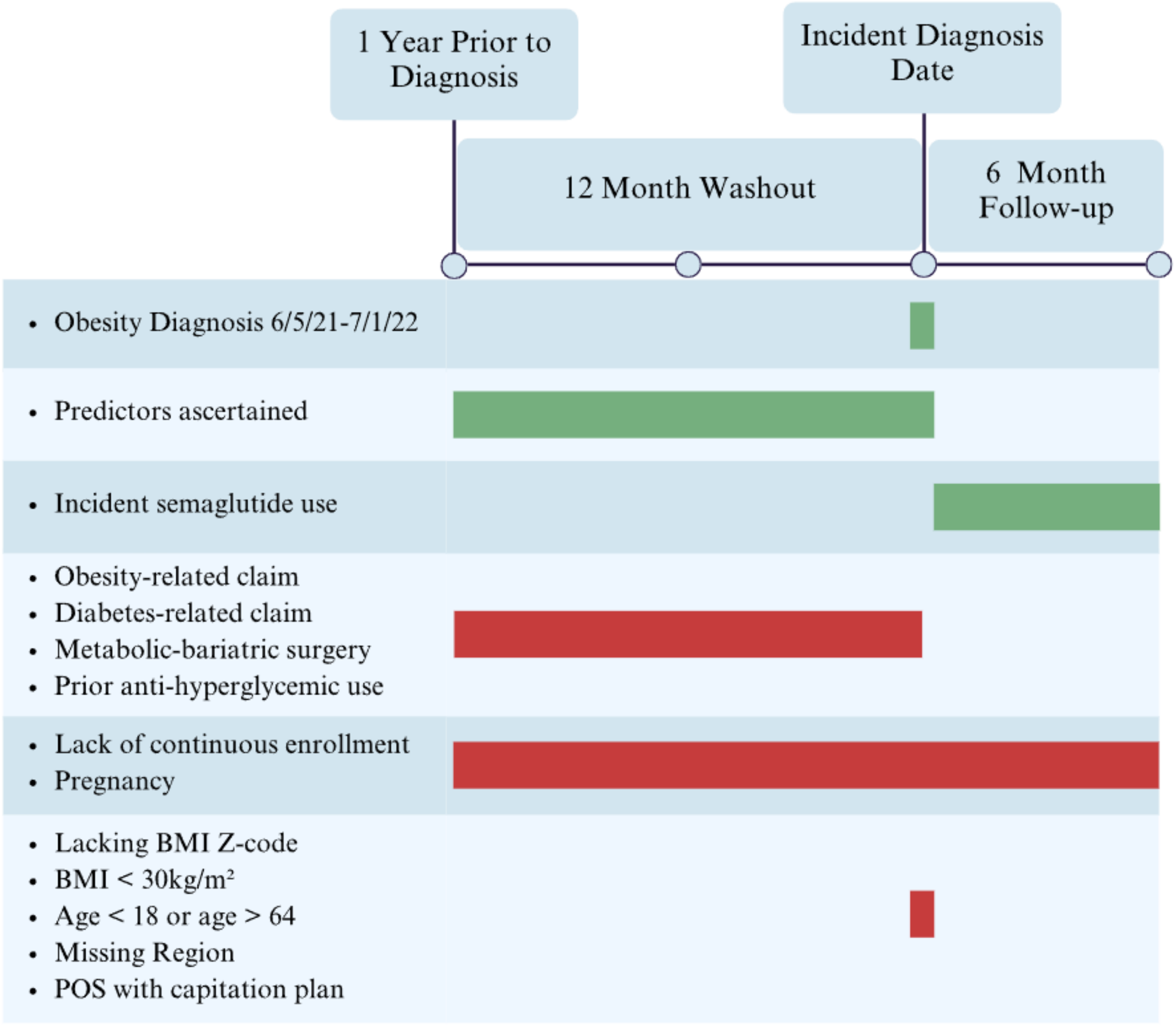

© 2025 Podolsky MI et al. JAMA Network Open.

eFigure 2. Trends in Semaglutide Initiation After Initial Obesity Diagnosis

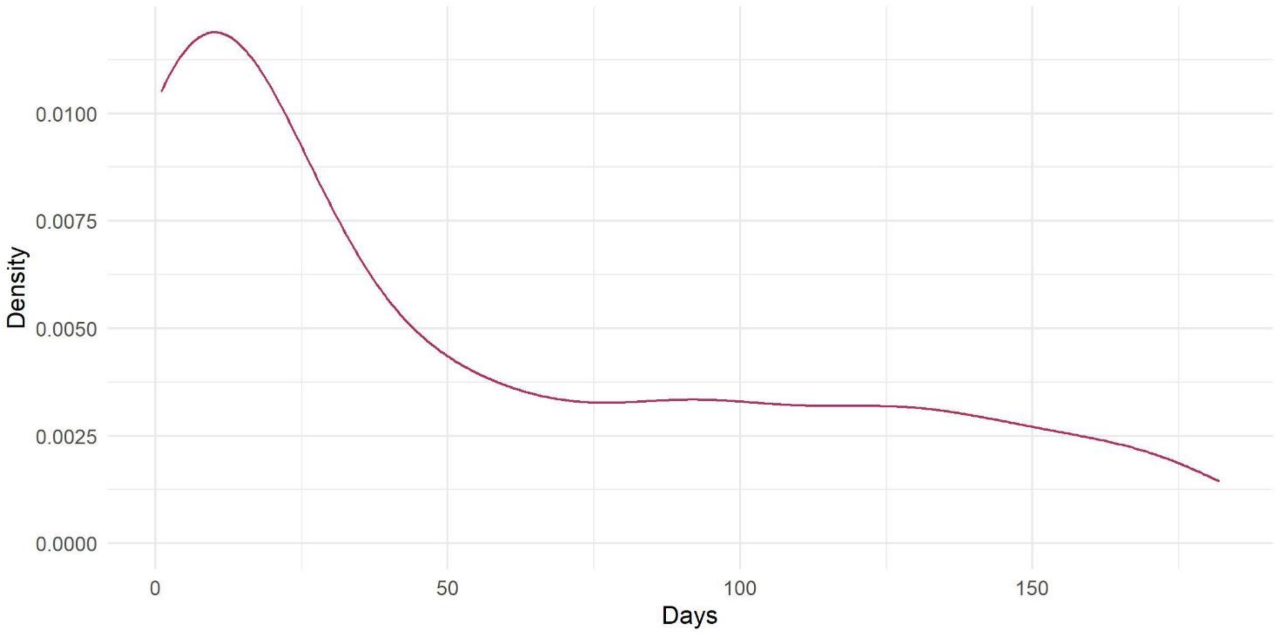

The time scale shown here is the number of days since first obesity-related inpatient or outpatient visit, as indicated by an E66- ICD-10 code. The density shown is the percentage of the total sample that initiated semaglutide on a given day.

© 2025 Podolsky MI et al. JAMA Network Open.

eTable. List of Factors Included in Model

CCSR Category Description	Therapeutic Class	Demographic Factor
Nutritional anemia	Antihistamines & Comb, NEC	Age
Hemolytic anemia	Amebicides, NEC	BMI Category
Aplastic anemia	Antihelmintic, NEC	Baseline Month
Acute posthemorrhagic anemia	Antibiot, Aminoglycosides	Region Northeast
Sickle cell trait/anemia	Antibiot, Antifungal	Region North Central
Coagulation and hemorrhagic disorders	Antibiot, Cephalosporin and Rel.	Region South
Diseases of white blood cells	Antibiot, B-lactam Antibiotics	Region West
Immunity disorders	Antibiot, Erythromycin & Macrolide	Sex
Postprocedural or postoperative complications of the spleen	Antibiot, Penicillins	Rural/Urban
Other specified and unspecified hematologic conditions	Antibiot, Tetracyclines	Relationship to Employee Employee
Chronic rheumatic heart disease	Antibiotics, Misc	Relationship to Employee Spouse
Acute rheumatic heart disease	Antituberculosis Agents, NEC	Relationship to Employee Child
Nonrheumatic and unspecified valve disorders	Antivirals, NEC	Full time status
Endocarditis and endocardial disease	Antimalarial Agents, NEC	Industry Manufacturing, Durable Goods
Myocarditis and cardiomyopathy	Quinolones, NEC	Industry Manufacturing, Nondurable Goods
Pericarditis and pericardial disease	Sulfonamides & Comb, NEC	Industry Transportation, Communications, Utilities
Essential hypertension	Sulfones, NEC	Industry Retail Trade
Hypertension with complications and secondary hypertension	Urinary Anti-infectives, NEC	Industry Finance, Insurance, Real Estate
Acute myocardial infarction	Anti-infectives, Misc	Industry Services
Complications of acute myocardial infarction	Parasympathomimetic, NEC	Industry Other
Coronary atherosclerosis and other heart disease	Anticholinergic, NEC	Industry Unknown
Nonspecific chest pain	Antichol/Antiparkinsonian Agents	Plan Type Missing
Acute pulmonary embolism	Antichol/Antimuscarinic/Antispas	Plan Type Basic/major medical or comprehensive
Pulmonary heart disease	Sympathomimetic Agents, NEC	Plan Type POS
Other and ill-defined heart disease	Sympatholytic Agents NEC	Plan Type EPO/HMO
Conduction disorders	Muscle Relax, Skeletal Central	Plan Type PPO
Cardiac dysrhythmias	Muscle Relax, Skeletal, Misc	Plan Type CDHP
Cardiac arrest and ventricular fibrillation	Vascular 5HT1 Agonist, NEC	Plan Type HDHP
Heart failure	Autonomic, Nicotine Preparations	
Cerebral infarction	Blood Derivatives, NEC	
Acute hemorrhagic cerebrovascular disease	Antianemic, Iron Preparations	
Sequela of hemorrhagic cerebrovascular disease	Coag/Anticoag, Anticoagulants	
Occlusion or stenosis of precerebral or cerebral arteries without infarction	Coag, Anticoag, Hemostatics	
Other and ill-defined cerebrovascular disease	Hematopoietic Agents, NEC	

© 2025 Podolsky MI et al. JAMA Network Open.

Sequela of cerebral infarction and other cerebrovascular disease	Hemorrheologic Agents, NEC	
Peripheral and visceral vascular disease	Thrombolytic Agents, NEC	
Arterial dissections	Antiplatelet Agents, NEC	
Gangrene	Cardiac Drugs. NEC	
Aortic; peripheral; and visceral artery aneurysms	Cardiac, ACE Inhibitors	
Aortic and peripheral arterial embolism or thrombosis	Cardiac, Cardiac Glycosides	
Hypotension	Cardiac, Antiarrhythmic Agents	
Other specified and unspecified circulatory disease	Cardiac, Alpha-Beta Blockers	
Acute phlebitis; thrombophlebitis and thromboembolism	Cardiac, Beta Blockers	
Chronic phlebitis; thrombophlebitis and thromboembolism	Cardiac, Calcium Channel	
Varicose veins of lower extremity	Antihyperlipidemic Drugs, NEC	
Postthrombotic syndrome and venous insufficiency/hypertension	Hypotensive Agents, NEC	
Vasculitis	Vasodilating Agents, NEC	
Postprocedural or postoperative circulatory system complication	Analg/Antipyr, Salicylates	
Other specified diseases of veins and lymphatics	Analg/Antipyr, Nonsteroid/Antiinflam	
Any dental condition including traumatic injury	Anal/Antipyr, Opiate Agonists	
Nontraumatic dental conditions	Anal/Antipyr, Opiate Part Agonist	
Caries, periodontitis, and other preventable dental conditions	Analgesics/Antipyretics, NEC	
Intestinal infection	Opiate Antagonists, NEC	
Disorders of teeth and gingiva	Anticonvulsants, Benzodiazepines	
Diseases of mouth; excluding dental	Anticonv, Hydantoin Derivatives	
Esophageal disorders	Anticonv, Succinimides	
Gastroduodenal ulcer	Anticonvulsants, Misc	
Gastrointestinal and biliary perforation	Psychother, Antidepressants	
Gastritis and duodenitis	Psychother, Tranq/Antipsychotics	
Other specified and unspecified disorders of stomach and duodenum	Stimulant, Amphetamine Type	
Appendicitis and other appendiceal conditions	Stimulant, Non-Amphetamine	
Abdominal hernia	ASH, Barbiturates	
Regional enteritis and ulcerative colitis	ASH, Benzodiazepines	
Intestinal obstruction and ileus	Anxiolytic/Sedative/Hypnotic NEC	
Diverticulosis and diverticulitis	Antimanic Agents, NEC	
Hemorrhoids	CNS Agents, Misc.	
Anal and rectal conditions	Contraceptive Cream/Foam/Devices	
Peritonitis and intra-abdominal abscess	Fluoride Preparations, NEC	
Biliary tract disease	Thyroid Function, NEC	
Hepatic failure	Roentgenography, NEC	

© 2025 Podolsky MI et al. JAMA Network Open.

Other specified and unspecified liver disease	Diagnostic Agents, Misc, NEC	
Pancreatic disorders (excluding diabetes)	Electrolytic/Caloric/Water, NEC	
Gastrointestinal hemorrhage	Acidifying Agents, NEC	
Noninfectious gastroenteritis	Alkalinizing Agents, NEC	
Noninfectious hepatitis	Ammonia Detoxicants, NEC	
Postprocedural or postoperative digestive system complication	Repl Preps, Calcium Supp	
Other specified and unspecified gastrointestinal disorders	Repl Preps, Magn Preps and Comb	
Otitis media	Repl Preps, Potassium Supp	
Diseases of middle ear and mastoid (except otitis media)	Repl Preps, Zinc Preps & Comb	
Diseases of inner ear and related conditions	Repl Preps, Sodium Chlor Preps	
Hearing loss	Replacement Preparations, Misc	
Postprocedural or postoperative ear and/or mastoid process complication	Potassium Removing Resins, NEC	
Other specified and unspecified disorders of the ear	Caloric Agents, Amino Acid Preps	
Thyroid disorders	Caloric/Nutrition/Dietary Misc	
Diabetes mellitus without complication	Diuretics, Loop Diuretics	
Diabetes mellitus with complication	Diuretics, Potassium-Sparing	
Diabetes mellitus, Type 1	Diuretics, Thiazides & related	
Diabetes mellitus, Type 2	Diuretics, Carb Anhydrase Inhib	
Diabetes mellitus, due to underlying condition, drug or chemical induced, or other specified type	Irrigating Solutions, NEC	
Nutritional deficiencies	Enzymes, NEC	
Malnutrition	Antitussives/Cold Comb, NEC	
Obesity	Expectorants/Cold Comb, NEC	
Disorders of lipid metabolism	Mucolytics, Cold Comb, NEC	
Fluid and electrolyte disorders	Cough/Cough/Cold Comb, NEC	
Cystic fibrosis	Antiinfect, Antibiotics, EENT	
Pituitary disorders	Antiinfect, Antivirals, EENT	
Postprocedural or postoperative endocrine or metabolic complication	Antiifect, Sulfonamides EENT	
Other specified and unspecified endocrine disorders	Antiinfectives, Misc EENT	
Other specified and unspecified nutritional and metabolic disorders	Antiinfect, Antiinflam EENT	
Sequela of malnutrition and other nutritional deficiencies	Antiinflam Agents EENT, NEC	
External cause codes: cut/pierce; initial encounter	Eyewash/Eyestrm/Lubr/Tear, NEC	
External cause codes: drowning/submersion; initial encounter	Miotics, EENT, NEC	
External cause codes: fall; initial encounter	Mydriatics, EENT, NEC	
External cause codes: fire/burn; initial encounter	Vasoconstrictors EENT, NEC	
External cause codes: firearm; initial encounter	Eye/Ear/Nose/Throat Misc, NEC	

© 2025 Podolsky MI et al. JAMA Network Open.

External cause codes: machinery; initial encounter	Antacids/Adsorbents & Comb, NEC	
External cause codes: motor vehicle traffic (MVT); initial encounter	Antidiarrhea Agents, NEC	
External cause codes: pedal cyclist; not MVT; initial encounter	Antiflatulents, NEC	
External cause codes: pedestrian; not MVT; initial encounter	Cath & Lax, Laxatives, Enemas	
External cause codes: transport; not MVT; initial encounter	Cath & Lax, Laxatives, Saline	
External cause codes: natural/environment; initial encounter	Cath & Lax, Laxatives, Stimulant	
External cause codes: bites; initial encounter	Cath & Lax, Laxatives, Stool Softeners	
External cause codes: overexertion; initial encounter	Cholelitholytic Agents, NEC	
External cause codes: poisoning by drug	Digestants & Comb, NEC	
External cause codes: poisoning by non-drug	Antiemetics, NEC	
External cause codes: struck by; against; initial encounter	Histamine (H2) Antagonists, NEC	
External cause codes: suffocation/inhalation; initial encounter	Gastrointestinal Drugs Misc, NEC	
External cause codes: other specified, classifiable and NEC; initial encounter	Hormones & Synthetics Subst, NEC	
External cause codes: unspecified mechanism	Adrenals & Comb, NEC	
External cause codes: intent of injury, accidental/unintentional	Androgens & Comb, NEC	
External cause codes: intent of injury, self-harm	Contraceptive, Oral Comb, NEC	
External cause codes: intent of injury, assault	Ovulation Stimulants, NEC	
External cause codes: intent of injury, undetermined	Estrogens & Comb, NEC	
External cause codes: intent of injury, legal intervention/war	Gonadotropins, NEC	
External cause codes: complications of medical and surgical care, initial encounter	Parathyroid Hormones, NEC	
External cause codes: activity codes	Pituitary Hormones, NEC	
External cause codes: place of occurrence of the external cause	Progestins, NEC	
External cause codes: evidence of alcohol involvement	Thy/Antithy, Thyroid Hormones	
External cause codes: subsequent encounter	Thy/Antithy, Antithyroid Agents	
External cause codes: sequela	Immunosuppressants, NEC	
Cornea and external disease	Anesthetics, Local	
Cataract and other lens disorders	Oxytocics, NEC	
Glaucoma	Serums/Toxoids/Vaccines, NEC	
Uveitis and ocular inflammation	Serums, NEC	
Retinal and vitreous conditions	Toxins, NEC	
Neuro-ophthalmology	Toxoids, NEC	
Strabismus	Vaccines, NEC	
Oculofacial plastics and orbital conditions	Antiinf S/MM, Antibiotics & Comb	
Refractive error	Antiinf S/MM, Antivirals & Comb	

© 2025 Podolsky MI et al. JAMA Network Open.

Blindness and vision defects	Antiinf S/MM, Antifungals & Comb	
Postprocedural or postoperative eye complication	Antiinf S/MM, Scabic/Pediculic	
Other specified eye disorders	Antiinf S/MM, Antiinf Local Misc	
Encounter for administrative purposes	Antiinflam S/MM Agnts & Comb, Misc	
Encounter for mental health services related to abuse	Antiprut/Local Anest S/MM, NEC	
Encounter for observation and examination for conditions ruled out (excludes infectious disease, neoplasm, mental disorders)	Cell Stim/Proliferant S/MM, NEC	
Encounter for prophylactic or other procedures	Emoll/Moist/Demul/Protect S/MM	
Encounter for prophylactic measures (excludes immunization)	Keratolytic Agents S/MM, NEC	
Encounter for antineoplastic therapies	Keratoplastic Agents S/MM, NEC	
Encounter for mental health conditions	S/MM Miscellaneous, NEC	
Neoplasm-related encounters	S/MM Misc, Astringents	
Implant, device or graft related encounter	S/MM, Soaps/Cleansers/Antiseptics	
Other aftercare encounter	S/MM, Skin and Wound Dress/Soaks	
Counseling related to sexual behavior or orientation	Depig/Pig/S/MM Depigment Agents	
Other specified encounters and counseling	Enzyme Preps, Topical S/MM, NEC	
Contraceptive and procreative management	Muscle Rel, Smooth-Genitour NEC	
Medical examination/evaluation	Muscle Rel, Smooth-Respiratr NEC	
Resistance to antimicrobial drugs	Vitamin A & Derivatives	
Exposure, encounters, screening or contact with infectious disease	Vitamin Bs & B Complex, NEC	
No immunization or underimmunization	Vitamin Bs w/Vitamin C, NEC	
Screening for neurocognitive or neurodevelopmental condition	Folic Acid & Derivatives, NEC	
Socioeconomic/psychosocial factors	Vitamin C & Bioflavanoids, NEC	
Lifestyle/life management factors	Vitamin D, NEC	
Personal/family history of disease	Multivit Prep, Multivit Plain	
Acquired absence of limb or organ	Multivit Prep, Multivit Iron	
Organ transplant status	Multivit Prep, Multivit Minerals	
Carrier status	Multivit Prep, Multivit Fluoride	
Other specified status	Multivit Prep, Multivit Prenatal	
Nephritis; nephrosis; renal sclerosis	Vitamins & Comb Misc, NEC	
Acute and unspecified renal failure	Unclassified Agents, NEC	
Chronic kidney disease	Antigout Agents, NEC	
Urinary tract infections	Mast Cell Stabilizers, NEC	
Calculus of urinary tract	Devices and Non-Drug Items, NEC	
Other specified and unspecified diseases of kidney and ureters	Pharmaceutical Aids/Adjuv, NEC	
Other specified and unspecified diseases of bladder and urethra	Antiallergic Agents	
Urinary incontinence	Phosphorus Removing Agents, NEC	
Hematuria	Antineoplastics S/MM, NEC	

© 2025 Podolsky MI et al. JAMA Network Open.

Proteinuria	Gonadotrop Rel Horm Antagonist	
Vesicoureteral reflux	Leukotriene Modifiers	
Hyperplasia of prostate	Phosphodiesterase Inhibitors	
Inflammatory conditions of male genital organs	Biological Response Modifiers	
Erectile dysfunction	Enzyme Inhibitors	
Male infertility	Bone Resorption Inhibitors	
Other specified male genital disorders	Interferons, Antineoplastic	
Nonmalignant breast conditions	Chemotherapy	
Inflammatory diseases of female pelvic organs	Hormone-Modifying Therapy	
Endometriosis	Molecular Targeted Therapy	
Prolapse of female genital organs	Antineoplastic Agent, Misc.	
Menstrual disorders	COMT Inhibitors	
Benign ovarian cyst	Per-Act Mu Op Rcp Ant (PAMORA)	
Menopausal disorders	Other/unavailable	
Female infertility		
Other specified female genital disorders		
Postprocedural or postoperative genitourinary system complication		
Tuberculosis		
Septicemia		
Bacterial infections		
Fungal infections		
Foodborne intoxications		
HIV infection		
Hepatitis		
Viral infection		
Parasitic, other specified and unspecified infections		
Sexually transmitted infections (excluding HIV and hepatitis)		
Sequela of specified infectious disease conditions		
Coronavirus disease – 2019 (COVID-19)		
Fracture of head and neck, initial encounter		
Fracture of the spine and back, initial encounter		
Fracture of torso, initial encounter		
Fracture of the upper limb, initial encounter		
Fracture of the lower limb (except hip), initial encounter		
Fracture of the neck of the femur (hip), initial encounter		
Dislocations, initial encounter		
Traumatic brain injury (TBI); concussion, initial encounter		
Spinal cord injury (SCI), initial encounter		

© 2025 Podolsky MI et al. JAMA Network Open.

Internal organ injury, initial encounter		
Open wounds of head and neck, initial encounter		
Open wounds to limbs, initial encounter		
Open wounds of trunk, initial encounter		
Amputation of a limb, initial encounter		
Amputation of other body parts, initial encounter		
Injury to blood vessels, initial encounter		
Superficial injury; contusion, initial encounter		
Crushing injury, initial encounter		
Burn and corrosion, initial encounter		
Effect of foreign body entering opening, initial encounter		
Effect of other external causes, initial encounter		
Poisoning by drugs, initial encounter		
Toxic effects, initial encounter		
Sprains and strains, initial encounter		
Injury to nerves, muscles and tendons, initial encounter		
Other specified injury		
Other unspecified injury		
Adverse effects of drugs and medicaments, initial encounter		
Underdosing of drugs and medicaments, initial encounter		
Drug induced or toxic related condition		
Allergic reactions		
Maltreatment/abuse		
Complication of cardiovascular device, implant or graft, initial encounter		
Complication of genitourinary device, implant or graft, initial encounter		
Complication of internal orthopedic device or implant, initial encounter		
Complication of transplanted organs or tissue, initial encounter		
Complication of other surgical or medical care, injury, initial encounter		
Fracture of head and neck, subsequent encounter		
Fracture of the spine and back, subsequent encounter		
Fracture of torso, subsequent encounter		
Fracture of the upper limb, subsequent encounter		
Fracture of lower limb (except hip), subsequent encounter		
Fracture of the neck of the femur (hip), subsequent encounter		
Dislocations, subsequent encounter		

© 2025 Podolsky MI et al. JAMA Network Open.

Traumatic brain injury (TBI); concussion, subsequent encounter		
Spinal cord injury (SCI), subsequent encounter		
Internal organ injury, subsequent encounter		
Open wounds of head and neck, subsequent encounter		
Open wounds to limbs, subsequent encounter		
Open wounds of trunk, subsequent encounter		
Amputation of a limb, subsequent encounter		
Injury to blood vessels, subsequent encounter		
Superficial injury; contusion, subsequent encounter		
Crushing injury, subsequent encounter		
Burns and corrosion, subsequent encounter		
Effect of foreign body entering opening, subsequent encounter		
Effect of other external causes, subsequent encounter		
Poisoning by drugs, subsequent encounter		
Toxic effects, subsequent encounter		
Sprains and strains, subsequent encounter		
Injury to nerves, muscles and tendons, subsequent encounter		
Other specified injury, subsequent encounter		
Other unspecified injuries, subsequent encounter		
Adverse effects of drugs and medicaments, subsequent encounter		
Underdosing of drugs and medicaments, subsequent encounter		
Allergic reactions, subsequent encounter		
Maltreatment/abuse, subsequent encounter		
Complication of cardiovascular device, implant or graft, subsequent encounter		
Complication of genitourinary device, implant or graft, subsequent encounter		
Complication of internal orthopedic device or implant, subsequent encounter		
Complication of other surgical or medical care, injury, subsequent encounter		
Injury, sequela		
Effect of other external causes, sequela		
Poisoning/toxic effect/adverse effects/underdosing, sequela		
Complication, sequela		
Cardiac and circulatory congenital anomalies		
Digestive congenital anomalies		
Genitourinary congenital anomalies		

© 2025 Podolsky MI et al. JAMA Network Open.

Nervous system congenital anomalies		
Congenital malformations of eye, ear, face, neck		
Cleft lip or palate		
Respiratory congenital malformations		
Musculoskeletal congenital conditions		
Chromosomal abnormalities		
Other specified and unspecified congenital anomalies		
Schizophrenia spectrum and other psychotic disorders		
Depressive disorders		
Bipolar and related disorders		
Other specified and unspecified mood disorders		
Anxiety and fear-related disorders		
Obsessive-compulsive and related disorders		
Trauma- and stressor-related disorders		
Disruptive, impulse-control and conduct disorders		
Personality disorders		
Feeding and eating disorders		
Somatic disorders		
Suicidal ideation/attempt/intentional self-harm		
Miscellaneous mental and behavioral disorders/conditions		
Neurodevelopmental disorders		
Alcohol-related disorders		
Opioid-related disorders		
Cannabis-related disorders		
Sedative-related disorders		
Stimulant-related disorders		
Hallucinogen-related disorders		
Inhalant-related disorders		
Tobacco-related disorders		
Other specified substance-related disorders		
Mental and substance use disorders in remission		
Suicide attempt/intentional self-harm; subsequent encounter		
Opioid-related disorders; subsequent encounter		
Stimulant-related disorders; subsequent encounter		
Cannabis-related disorders; subsequent encounter		
Hallucinogen-related disorders; subsequent encounter		
Sedative-related disorders; subsequent encounter		

© 2025 Podolsky MI et al. JAMA Network Open.

Mental and substance use disorders; sequela		
Infective arthritis		
Osteomyelitis		
Rheumatoid arthritis and related disease		
Juvenile arthritis		
Other specified chronic arthropathy		
Osteoarthritis		
Other specified joint disorders		
Immune-mediated/reactive arthropathies		
Tendon and synovial disorders		
Musculoskeletal pain, not low back pain		
Spondylopathies/spondyloarthropathy (including infective)		
Biomechanical lesions		
Osteoporosis		
Pathological fracture, initial encounter		
Pathological fracture, subsequent encounter		
Stress fracture, initial encounter		
Stress fracture, subsequent encounter		
Atypical fracture, initial encounter		
Atypical fracture, subsequent encounter		
Pathological, stress and atypical fractures, sequela		
Acquired foot deformities		
Scoliosis and other postural dorsopathic deformities		
Acquired deformities (excluding foot)		
Systemic lupus erythematosus and connective tissue disorders		
Other specified connective tissue disease		
Muscle disorders		
Musculoskeletal abscess		
Other specified bone disease and musculoskeletal deformities		
Disorders of jaw		
Aseptic necrosis and osteonecrosis		
Traumatic arthropathy		
Neurogenic/neuropathic arthropathy		
Gout		
Crystal arthropathies (excluding gout)		
Osteomalacia		
Autoinflammatory syndromes		
Postprocedural or postoperative musculoskeletal system complication		
Low back pain		
Head and neck cancers - eye		

© 2025 Podolsky MI et al. JAMA Network Open.

Head and neck cancers - lip and oral cavity		
Head and neck cancers - throat		
Head and neck cancers - salivary gland		
Head and neck cancers - nasopharyngeal		
Head and neck cancers - hypopharyngeal		
Head and neck cancers - pharyngeal		
Head and neck cancers - laryngeal		
Head and neck cancers - tonsils		
Head and neck cancers - all other types		
Cardiac cancers		
Gastrointestinal cancers - esophagus		
Gastrointestinal cancers - stomach		
Gastrointestinal cancers - small intestine		
Gastrointestinal cancers - colorectal		
Gastrointestinal cancers - anus		
Gastrointestinal cancers - liver		
Gastrointestinal cancers - bile duct		
Gastrointestinal cancers - gallbladder		
Gastrointestinal cancers - peritoneum		
Gastrointestinal cancers - all other types		
Respiratory cancers		
Bone cancer		
Sarcoma		
Skin cancers - melanoma		
Skin cancers - basal cell carcinoma		
Skin cancers - squamous cell carcinoma		
Skin cancers - all other types		
Breast cancer - ductal carcinoma in situ (DCIS)		
Breast cancer - all other types		
Female reproductive system cancers - uterus		
Female reproductive system cancers - cervix		
Female reproductive system cancers - ovary		
Female reproductive system cancers - fallopian tube		
Female reproductive system cancers - endometrium		
Female reproductive system cancers - vulva		
Female reproductive system cancers - vagina		
Female reproductive system cancers - all other types		
Male reproductive system cancers - prostate		
Male reproductive system cancers - testis		

© 2025 Podolsky MI et al. JAMA Network Open.

Male reproductive system cancers - penis		
Male reproductive system cancers - all other types		
Urinary system cancers - bladder		
Urinary system cancers - ureter and renal pelvis		
Urinary system cancers - kidney		
Urinary system cancers - urethra		
Urinary system cancers - all other types		
Nervous system cancers - brain		
Nervous system cancers - all other types		
Endocrine system cancers - thyroid		
Endocrine system cancers - pancreas		
Endocrine system cancers - thymus		
Endocrine system cancers - adrenocortical		
Endocrine system cancers - parathyroid		
Endocrine system cancers - pituitary gland		
Endocrine system cancers - all other types		
Hodgkin lymphoma		
Non-Hodgkin lymphoma		
Leukemia - acute lymphoblastic leukemia (ALL)		
Leukemia - acute myeloid leukemia (AML)		
Leukemia - chronic lymphocytic leukemia (CLL)		
Leukemia - chronic myeloid leukemia (CML)		
Leukemia - hairy cell		
Leukemia - all other types		
Multiple myeloma		
Malignant neuroendocrine tumors		
Mesothelioma		
Myelodysplastic syndrome (MDS)		
Cancer of other sites		
Secondary malignancies		
Malignant neoplasm, unspecified		
Neoplasms of unspecified nature or uncertain behavior		
Benign neoplasms		
Conditions due to neoplasm or the treatment of neoplasm		
Meningitis		
Encephalitis		
Other specified CNS infection and poliomyelitis		
Parkinson`s disease		
Multiple sclerosis		

© 2025 Podolsky MI et al. JAMA Network Open.

Other nervous system disorders (often hereditary or degenerative)		
Cerebral palsy		
Paralysis (other than cerebral palsy)		
Epilepsy; convulsions		
Headache; including migraine		
Neurocognitive disorders		
Transient cerebral ischemia		
Coma; stupor; and brain damage		
CNS abscess		
Polyneuropathies		
Sleep wake disorders		
Nerve and nerve root disorders		
Myopathies		
Nervous system pain and pain syndromes		
Other nervous system disorders (neither hereditary nor degenerative)		
Postprocedural or postoperative nervous system complication		
Sequela of specified nervous system conditions		
Liveborn		
Short gestation; low birth weight; and fetal growth retardation		
Neonatal acidemia and hypoxia		
Neonatal cerebral disorders		
Respiratory distress syndrome		
Respiratory perinatal condition		
Hemolytic jaundice and perinatal jaundice		
Birth trauma		
Perinatal infections		
Newborn affected by maternal conditions or complications of labor/delivery		
Hemorrhagic and hematologic disorders of newborn		
Neonatal digestive and feeding disorders		
Other specified and unspecified perinatal conditions		
Neonatal abstinence syndrome		
Fetal alcohol syndrome		
Antenatal screening		
Gestational weeks		
Spontaneous abortion and complications of spontaneous abortion		
Induced abortion and complications of termination of pregnancy		
Ectopic pregnancy and complications of ectopic pregnancy		

© 2025 Podolsky MI et al. JAMA Network Open.

Molar pregnancy and other abnormal products of conception		
Complications following ectopic and/or molar pregnancy		
Supervision of high-risk pregnancy		
Early, first or unspecified trimester hemorrhage		
Hemorrhage after first trimester		
Early or threatened labor		
Multiple gestation		
Maternal care related to fetal conditions		
Polyhydramnios and other problems of amniotic cavity		
Obstetric history affecting care in pregnancy		
Previous C-section		
Maternal care for abnormality of pelvic organs		
Maternal care related to disorders of the placenta and placental implantation		
Diabetes or abnormal glucose tolerance complicating pregnancy; childbirth; or the puerperium		
Hypertension and hypertensive-related conditions complicating pregnancy; childbirth; and the puerperium		
Maternal intrauterine infection		
Prolonged pregnancy		
Complications specified during childbirth		
Malposition, disproportion or other labor complications		
Anesthesia complications during pregnancy		
OB-related trauma to perineum and vulva		
Complications specified during the puerperium		
Other specified complications in pregnancy		
Uncomplicated pregnancy, delivery or puerperium		
Maternal outcome of delivery		
Sinusitis		
Pneumonia (except that caused by tuberculosis)		
Influenza		
Acute and chronic tonsillitis		
Acute bronchitis		
Other specified upper respiratory infections		
Other specified and unspecified upper respiratory disease		
Chronic obstructive pulmonary disease and bronchiectasis		
Asthma		

© 2025 Podolsky MI et al. JAMA Network Open.

Aspiration pneumonitis		
Pleurisy, pleural effusion and pulmonary collapse		
Respiratory failure; insufficiency; arrest		
Lung disease due to external agents		
Pneumothorax		
Mediastinal disorders		
Other specified and unspecified lower respiratory disease		
Postprocedural or postoperative respiratory system complication		
Skin and subcutaneous tissue infections		
Other specified inflammatory condition of skin		
Pressure ulcer of skin		
Non-pressure ulcer of skin		
Contact dermatitis		
Postprocedural or postoperative skin complication		
Other specified and unspecified skin disorders		
Syncope		
Fever		
Shock		
Nausea and vomiting		
Dysphagia		
Abdominal pain and other digestive/abdomen signs and symptoms		
Malaise and fatigue		
Symptoms of mental and substance use conditions		
Abnormal findings related to substance use		
Nervous system signs and symptoms		
Genitourinary signs and symptoms		
Circulatory signs and symptoms		
Respiratory signs and symptoms		
Skin/Subcutaneous signs and symptoms		
General sensation/perception signs and symptoms		
Other general signs and symptoms		
Abnormal findings without diagnosis		
Used to indicate the diagnosis was invalid and could not be assigned a CCSR category		

© 2025 Podolsky MI et al. JAMA Network Open.

eFigure 3. CONSORT Diagram

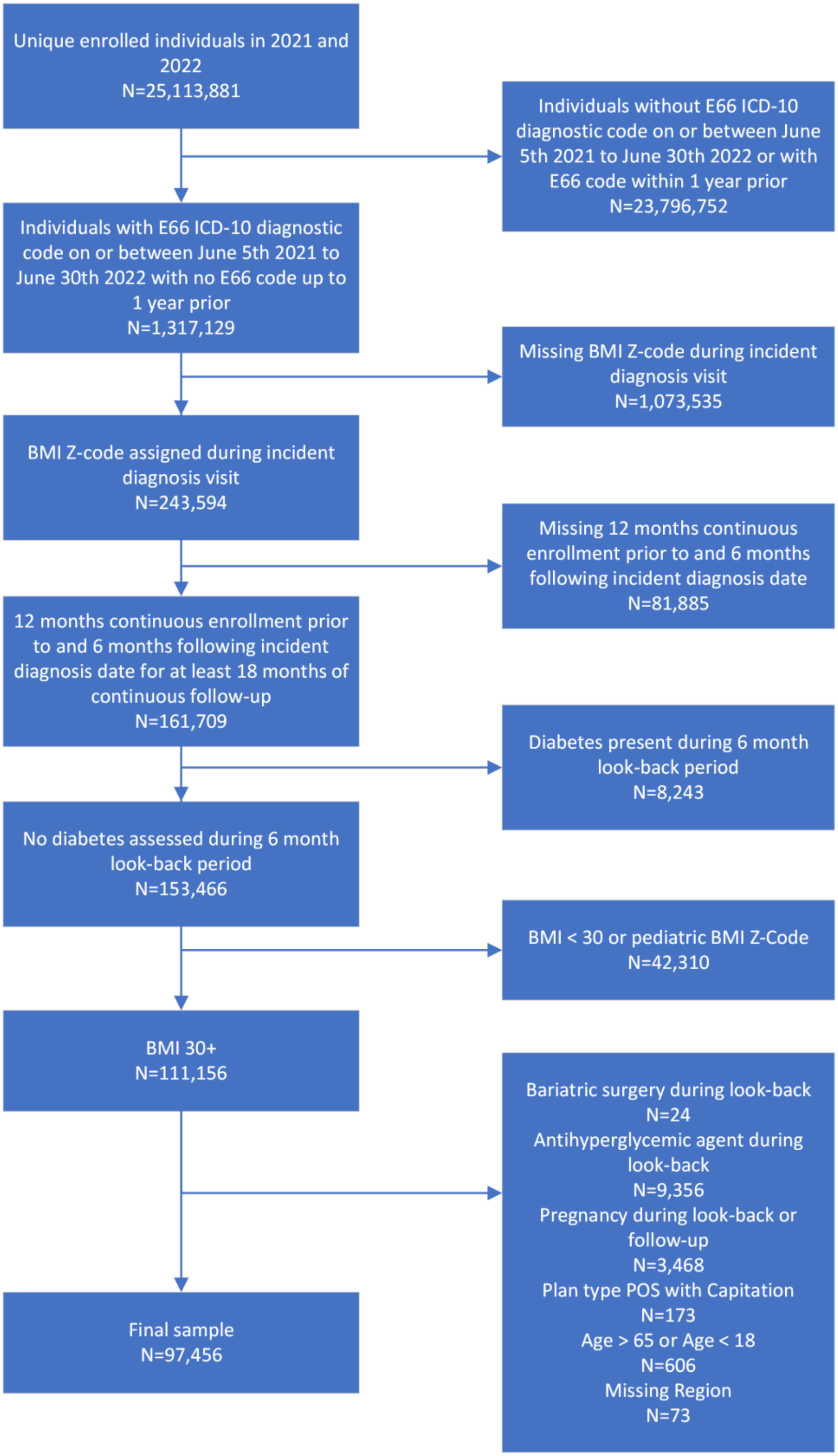

eFigure 4. Performance of 10-Fold Cross-Classified Random Forest Models for Estimating Semaglutide Initiation Among Individuals With Obesity Without Diabetes

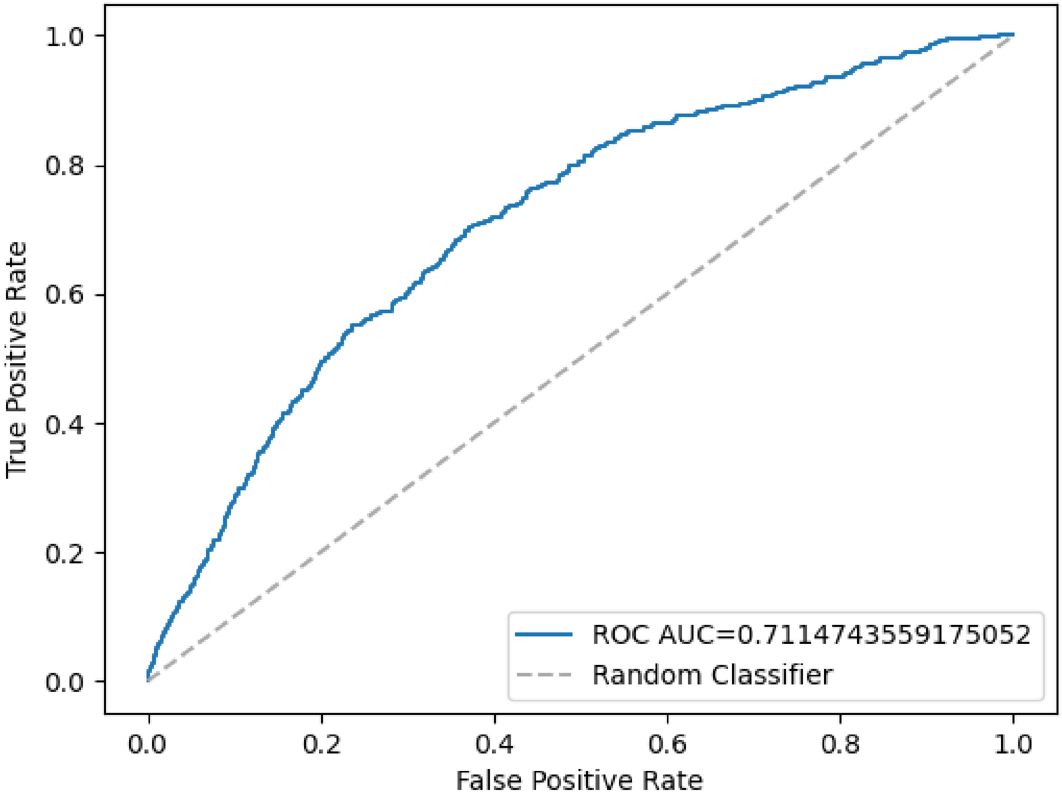

ROC AUC: Area under the receiver operating characteristic curve, an aggregate measure of model performance.

© 2025 Podolsky MI et al. JAMA Network Open.

eFigure 5. SHAP Plot for Dataset With Oversample of Positive Class

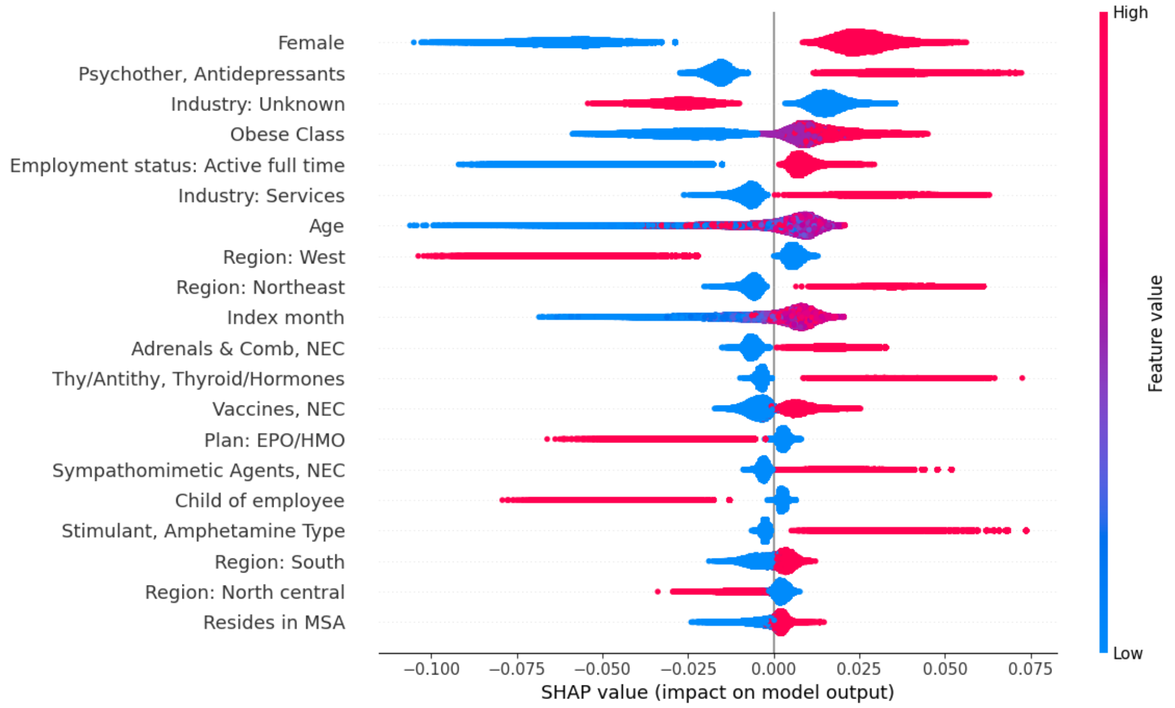

To address the classification issues of an imbalanced dataset, we took a random oversample of the positive class sample (people who received semaglutide) until we created a dataset where the outcome occurred for 50% of the sample. The model fit to this dataset identified largely the same top 20 important features. We repeated the process with an undersample of the negative class sample (people who did not receive semaglutide) and the outcome was similar.

© 2025 Podolsky MI et al. JAMA Network Open.

eFigure 6. SHAP Plot for Dataset With Oversample of Negative Class

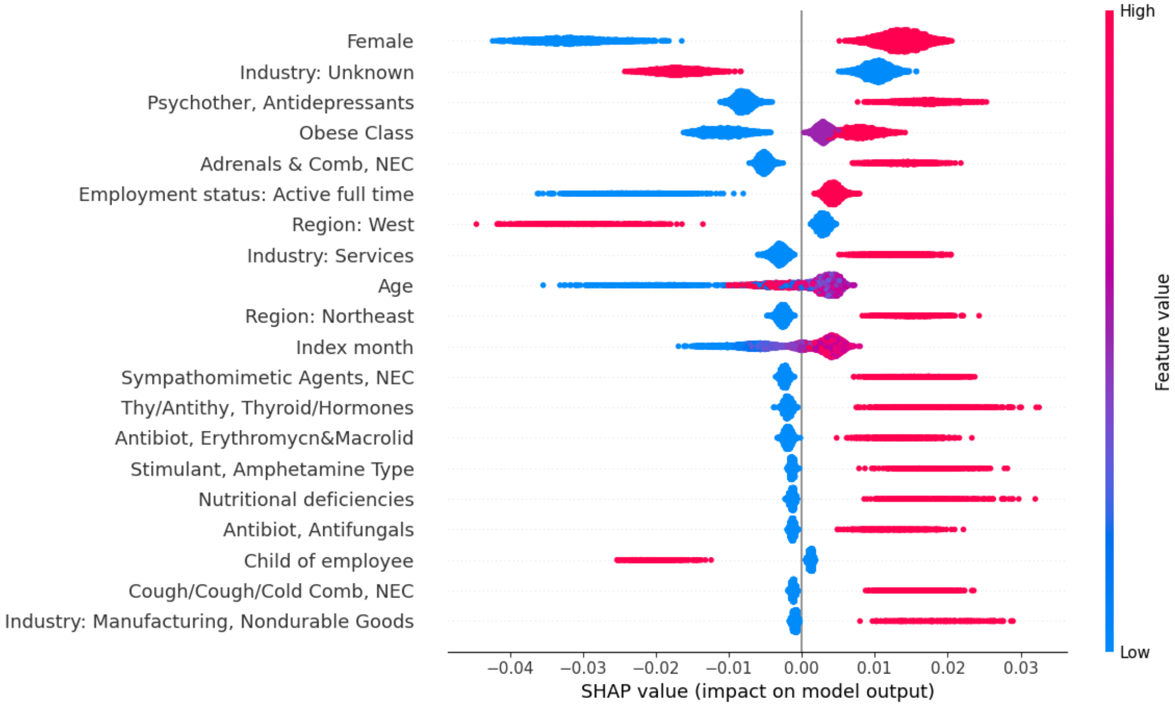

To address the classification issues of an imbalanced dataset, we took a random oversample of the positive class sample (people who received semaglutide) until we created a dataset where the outcome occurred for 50% of the sample. The model fit to this dataset identified largely the same top 20 important features. We repeated the process with an undersample of the negative class sample (people who did not receive semaglutide) and the outcome was similar.

© 2025 Podolsky MI et al. JAMA Network Open.

eTable 2: Mean and Scaled SHAP Values

	Description (Value)	Mean SHAP Value	Scaled SHAP Value (%)
Female	Female sex at baseline (0 = No, 1 = Yes)	0.00249	100.0
Psychother, Antidepressants	Prescribed medication in Redbook therapeutic class Psychother, Antidepressants during washout (0 = No, 1 = Yes)	0.00162	65.1
Industry: Unknown	Works in unknown industry at baseline (0 = No, 1 = Yes)	0.00161	64.7
Obese Class	Obese class category at baseline, based on WHO definitions (1 = 30.0-34.9, 2 = 35.0-39.9, 3 = 40.0+)	0.00117	47.2
Industry: Services	Works in services industry at baseline (0 = No, 1 = Yes)	0.00112	45.2
Employment status: Active full time	Actively employed full time at baseline (0 = No, 1 = Yes)	0.00095	38.2
Region: Northeast	Lives in region, Northeast, during baseline (0 = No, 1 = Yes)	0.00078	31.4
Age	Continuous age at baseline (18-64)	0.00067	27.1
Adrenals & Comb, NEC	Prescribed medication in Redbook therapeutic class Adrenals & Comb, NEC during washout (0 = No, 1 = Yes)	0.00061	24.3
Index month	Month of incident obesity visit (1 = June 2021, 2 = July 2021, 3 = August 2021, 4 = September 2021, 5 = October 2021, 6 = November 2021, 7 = December 2021, 8 = January 2022, 9 = February 2022, 10 = March 2022, 11 = April 2022, 12 = May 2022, 13 = June 2022)	0.00057	23.1
Thy/Antithy, Thyroid/Hormones	Prescribed medication in Redbook therapeutic class Thy/Antithy, Thyroid/Hormones during washout (0 = No, 1 = Yes)	0.00055	22.0
Anticonvulsants, Misc	Prescribed medication in Redbook therapeutic class Anticonvulsants, Misc during washout (0 = No, 1 = Yes)	0.00044	17.5
Sympathomimetic Agents, NEC	Prescribed medication in Redbook therapeutic class Sympathomimetic Agents, NEC during washout (0 = No, 1 = Yes)	0.00043	17.1
Vaccines, NEC	Prescribed medication in Redbook therapeutic class Vaccines, NEC during washout (0 = No, 1 = Yes)	0.00034	13.8
Stimulant, Amphetamine Type	Prescribed medication in Redbook therapeutic class Stimulant, Amphetamine Type during washout (0 = No, 1 = Yes)	0.00033	13.3
Other general signs and symptoms	Visit in CCSR category Other general signs and symptoms during washout (0 = No, 1 = Yes)	0.00032	13.0
Resides in MSA	Resides in MSA during baseline (0 = No, 1 = Yes)	0.00026	10.5
Region: South	Lives in region, South, during baseline (0 = No, 1 = Yes)	0.00026	10.5
Analg/Antipyr,Nonstr/Antiinflm	Prescribed medication in Redbook therapeutic class Analg/Antipyr,Nonstr/Antiinflm during washout (0 = No, 1 = Yes)	0.00022	9.0
Plan: PPO	Plan type PPO at baseline (0 = No, 1 = Yes)	0.00022	8.8

© 2025 Podolsky MI et al. JAMA Network Open.
