# Supplementary material for: Factors Associated With Semaglutide Initiation Among Adults With Obesity
Source: JAMA Netw Open. 2025 Jan 21;8(1):e2455222. doi: 10.1001/jamanetworkopen.2024.55222 (PMC11751746; doi:10.1001/jamanetworkopen.2024.55222)
Supplement: Supplement 2. — Data Sharing Statement [file jamanetwopen-e2455222-s002.pdf]

Data Sharing Statement

Podolsky. Factors Associated With Semaglutide Initiation Among Adults With Obesity. *JAMA Netw Open*. Published January 21, 2025. doi:10.1001/jamanetworkopen.2024.55222

Data
Data available: No

Additional Information
Explanation for why data not available: The data used in this study require a license to the Merative MarketScan Commercial Claims and Encounters Database
